# T and B Cell Composition and Cytokine Producing Capacity Before and After Bariatric Surgery

**DOI:** 10.3389/fimmu.2022.888278

**Published:** 2022-07-04

**Authors:** L. H. Wijngaarden, A. E. Taselaar, F. Nuijten, E. van der Harst, R. A. Klaassen, T. M. Kuijper, F. Jongbloed, G. Ambagtsheer, M. Klepper, J. N. M. IJzermans, R. W. F. de Bruin, N. H. R. Litjens

**Affiliations:** ^1^Department of Surgery, Erasmus MC, Erasmus MC Transplant Institute, University Medical Center, Rotterdam, Netherlands; ^2^Department of Surgery, Maasstad Hospital, Rotterdam, Netherlands; ^3^Maasstad Academy, Maasstad Hospital, Rotterdam, Netherlands; ^4^Department of Internal Medicine, Erasmus MC, University Medical Center, Rotterdam, Netherlands; ^5^Department of Internal Medicine, Erasmus MC Transplant Institute, Division Nephrology and Transplantation, Erasmus MC, University Medical Center, Rotterdam, Netherlands

**Keywords:** immune cell function, cytokine producing capacity, morbid obesity, bariatric surgery, T cells, B cells Immune function and bariatric surgery

## Abstract

Morbid obesity is associated with a chronic state of low-grade inflammation, which may lead to accelerated differentiation of T and B cells. These differentiated immune cells are strongly cytotoxic and have an increased pro-inflammatory cytokine producing capacity. Furthermore, the anti-inflammatory function of the T and B cells decreases. The aim of this study was to evaluate the effect of morbid obesity on the subset profile and cytokine producing capacity of T and B cells. Subsequently, we assessed whether bariatric surgery affected the subset profile and cytokine producing capacity of these cells. We determined the proportion of T and B cell subsets and their cytokine producing capacity in peripheral blood collected from 23 morbidly obese patients before and three months after bariatric surgery using flow-cytometry. We compared this with the results of 25 lean controls. Both CD4+ and CD8+ T cells showed a more differentiated subset profile in morbidly obese patients as compared to lean controls, which was not recovered three months after bariatric surgery. The B cell composition of morbidly obese patients after bariatric surgery adjusted towards the profile of lean controls. However, the IL-2 and IFN-γ producing capacity of CD8+ T cells and the IL-2, IFN-γ, TNF-α and IL-10 producing capacity of B cells was not restored three months after bariatric surgery. In conclusion, the data suggest that the immune system has the capacity to recover from the detrimental effects of morbid obesity within three months after bariatric surgery in terms of cell composition; however, this was not seen in terms of cytokine producing capacity. The full restoration of the immune system after bariatric surgery may thus take longer.

## Introduction

Morbid obesity increases the risk for the development of obesity-related comorbidities, such as hypertension, type 2 diabetes mellitus (T2D) and cancer (1, 2). Morbid obesity is defined as a Body Mass Index (BMI) ≥35 kg/m^2^ with the presence of at least one obesity-related comorbidity or a BMI ≥40 kg/m^2^ either with or without the presence of obesity-related comorbidities. Furthermore, morbid obesity is associated with a chronic state of low-grade inflammation (3, 4). The high number of adipocytes in white adipose tissue of morbidly obese individuals secrete pro-inflammatory cytokines (such as tumor necrosis factor-alpha (TNF-α), interferon gamma (IFN-γ) and interleukins (IL-) 2 and 6), which may be related to this systemic inflammation (5). This is more pronounced in morbidly obese individuals with metabolic syndrome (MetS), which is characterized by three or more of the following symptoms: dyslipidemia, hypercholesterolemia, dysglycemia, an elevated blood pressure, and an increased abdominal waist circumference (6, 7). This pro-inflammatory environment may lead to a shift in immune cell composition and immune function in morbidly obese patients as compared to lean individuals (8, 9). Morbid obesity has been shown to decrease B cell function in humans, impairs B cell responses to infections and vaccines (10, 11) and leads to loss of T cell regulatory mechanisms (12). Also, presence of epithelial signals regulating the adipose tissue immunity may be altered in morbidly obese individuals (13).

Some T cells, identified as cluster of differentiation (CD) 3+CD4+ T cells, are referred to as T helper (Th) cells. Within this Th cell subset, different phenotypes can be distinguished in association with their function, e.g. Th1 are cells known to produce pro-inflammatory (type 1) cytokines such as IFN-γ and/or TNF-α, whereas Th2 cells are known for their production of anti-inflammatory cytokines such as IL-4 and/or IL-5. Aside from these Th cells, cytotoxic T (Tc) cells can be identified as CD3+CD8+ T cells.

T cells leave the thymus as naive, antigen-inexperienced T cells. Upon encounter of antigens presented by antigen presenting cells, naive T cells will differentiate into effector T cells. Eventually, only a fraction of these will develop into memory T cells. These different subsets can be dissected on the basis of expression of chemokine C-C motif receptor 7 (CCR7) and an isoform of CD45, being CD45RO (14). CCR7 is a chemokine receptor that when expressed on T cells allows them to migrate to secondary lymphoid organs. CD45RO is expressed by memory T cells. Naive T cells are CD45RO-CCR7+. Central memory (CM) T cells are CD45RO+ CCR7+ and are able to home into lymphoid tissues. Effector memory (EM) T cells are CD45RO+ CCR7- and they exert direct effector functions. Finally, the more terminally differentiated effector memory re-expressing CD45RA+ (hence termed EMRA), are CD45RO-CCR7- and they are high in effector function.

In the adaptive immune system, shifting of the T cell subset composition towards a more differentiated profile has been reported as a consequence of morbid obesity. This shifting typically shows similarities with the T cell composition of elderly individuals (8, 15, 16). A shift towards more differentiated memory T cells, such as EMRA T cells and CD28^null^ T cells, has been described (17, 18). These cells produce increased amounts of pro-inflammatory cytokines IFN-γ and TNF-α (19, 20), and decreased levels of IL-2 (21).

Similar changes in subset composition have been described in B cell populations, in which a more differentiated B cell profile is seen in morbidly obese individuals (22, 23). An increase in double negative (DN) B cells has been described in morbidly obese individuals, which is comparable to the cell subset composition found in elderly individuals (10, 22). Additionally, a change in B cell function in morbidly obese individuals has been described. Several studies have reported an increased production of pro-inflammatory cytokines such as IL-6, TNF-α and IFN-γ and a decrease in the production of the anti-inflammatory cytokine IL-10 (10, 24).

These dysfunctional T and B cells in morbidly obese individuals have several clinical consequences. The enhanced TNF-α and IFN-γ production in morbidly obese individuals leads to insulin resistance, causing a higher risk of T2D development (25–27). Additionally, as the IL-10 production is decreased in morbidly obese individuals, the IL-10 regulation of insulin sensitivity is lowered. This consequently leads to a higher risk of T2D development as well (28). Another clinical consequence of the chronic state of low-grade inflammation is the development of cardiovascular pathology. The increased production of TNF-α and IFN-γ by CD4+ and CD8+ T cells leads to atherosclerosis and hypertension, eventually resulting in cardiovascular diseases (29, 30). Subsequently, the lower levels of IL-2 lead to a decreased regulatory T cell function, which contributes to a persisting inflammation (21). Moreover, the decreased IL-2 producing capacity of both T and B cells in morbidly individuals leads to a decreased humoral response to vaccinations (10, 31).

Bariatric surgery is regarded an effective treatment for morbid obesity, resulting in significant weight loss and improving, or even resolving, obesity-related comorbidities (32–35). Only a few studies have been performed to assess the effects of bariatric surgery on T and B cell function. One study found an increase in IL-10 production by B cells in peripheral blood after laparoscopic Roux-en-Y gastric bypass (LRYGB) (36). An other study found that the number of T cells did not differ after LRYGB, although the cytokine producing capacity of the T cells did change after LRYGB (37). This resulted in a decreased IFN-γ, IL-2, IL-4 and IL-17 secretion by T cells and an increased IL-10 secretion by B cells. To our knowledge, previous studies to assess the T and B cell function have shown contradictory results, and the cytokine producing capacity of the T and B cells has not been compared to that of lean controls.

Therefore, the aim of this study was to investigate the T and B cell composition and cytokine producing capacity of morbidly obese patients and lean controls, and to study the effect of bariatric surgery on T and B cell cytokine producing capacity. Our hypothesis is that bariatric surgery decreases the pro-inflammatory environment and restores the cytokine production by T and B cells to the level of cytokine production of lean controls.

## Methods

### Patient Selection

Morbidly obese patients who were scheduled for a laparoscopic Roux-en-Y gastric bypass (LRYGB) or laparoscopic sleeve gastrectomy (LSG) between March 2014 and August 2015 in the Maasstad Hospital, Rotterdam, the Netherlands, were invited to participate in this non-randomized prospective cohort study. To be eligible for LRYGB or LSG, patients had to fulfil the criteria for bariatric surgery of the International Federation for the Surgery of Obesity and Metabolic Disorders (IFSO). Patients were excluded if their morbid obesity was caused by genetic defects or if they had previous bariatric surgery in their medical history. Medical history of eligible patients was checked on any abnormalities related to infections or immune status, including the use of corticosteroids. This was not the case. All patients gave written informed consent before inclusion.

Blood donors at the Sanquin blood bank were invited to participate in this study as lean, healthy controls. Patients with a recent history of abnormalities related to infections or immune status, including the use of immunosuppressive medications are not allowed to donate blood. The lean controls were aged between 18 to 65 years. Controls with a BMI > 30 kg/m^2^ and/or with the presence of metabolic syndrome were excluded from this study. Lean controls were informed about the study and were asked if two blood samples of 10mL each could be collected for this study. Oral informed consent was given to Sanquin blood bank during registration for blood donation. Blood samples were obtained between December 2018 and April 2019.

The local medical ethical committee (MEC) approved the study (MEC number: MEC-2018-06 for lean controls and MEC 2012-51 for morbidly obese patients). All participants of this study gave written or oral informed consent, as approved by the medical ethical committee. This study was conducted in accordance with the Declaration of Helsinki and the Declaration of Istanbul and in compliance with the International Conference on Harmonization/Good Clinical Practice regulations.

### Metabolic Syndrome

Metabolic syndrome was defined as the presence of at least three of the following symptoms (6):

1. Fasting blood glucose ≥ 5.6 mmol/L (100 mg/dL) or drug treatment for elevated blood glucose.
2. HDL cholesterol < 1.0 mmol/L (40 mg/dL) in men, < 1.3 mmol/L (50 mg/dL) in women or drug treatment for low HDL cholesterol.
3. Blood triglycerides ≥ 1.7 mmol/L (150 mg/dL) or drug treatment for elevated triglycerides.
4. Waist circumference ≥ 102 cm for men or ≥ 88 cm for women.
5. Blood pressure ≥ 130 mmHg systolic or ≥ 85 mmHg diastolic or antihypertensive drug treatment.

### Surgical Procedures

In this study, morbidly obese patients underwent either LRYGB or LSG (38). In the LRYGB procedure, first a gastric pouch with a volume of 25-30 cm^3^ was created using an Endostapler (Medtronic, Minneapolis, MN). Next, a biliopancreatic limb was measured 50 cm distal from the ligament of Treitz and stapled to the gastric pouch with an Endostapler, creating the posterior wall of the gastrojejunostomy. A continuous absorbable suture was used to close the anterior aspect of the gastrojejunostomy. A side-to-side jejunojejunostomy with an alimentary limb of 150 cm was created with an Endostapler and a continuous absorbable suture. Hereafter, a transection between both anastomoses was performed (39).

During LSG, a tubular sleeve was created using a 35 Fr bougie. The greater curvature was dissected starting 4-5 cm from the pylorus and up to the angle of His and was then removed using an endobag (40).

### Blood Collection

In morbidly obese patients, blood was obtained prior to surgery to determine the immune status. Three months postoperatively, blood collection took place during a routine outpatient clinic visit. Blood was also collected from lean controls during their visit at the Blood bank. Blood was collected in 10.0 mL BD Lithium-Heparin tubes (Franklin Lakes, NJ, USA), with a maximum of two tubes per time point.

### CMV Seropositivity

CMV seropositivity is associated with age-related changes in the circulating T cell compartment, such as an increased CD8+ T cell differentiation status and decreased CD4+/CD8+ T cell ratio (41, 42). To avoid confounding, CMV infection status was assessed in all participants at the diagnostic Department of Virology of Erasmus University Medical Center by determining the presence of plasma IgG antibodies to CMV using an enzyme immunoassay (Biomerieux, VIDAS, Lyon, France). An outcome of ≥ 6 arbitrary units per mL (AU/mL) was considered positive (43).

### PBMCs Isolation

Ficoll™ gradient centrifugation was used to isolate peripheral blood mononuclear cells (PBMCs) from heparinized blood samples, as described in detail by Litjens et al. (31). After isolation, samples were stored in liquid nitrogen with 10x10^6^ cells per vial. A vial of PBMCs was thawed at 37°C and added dropwise to a mixture containing 5 mL DNAse medium and 1 mL of normal human serum (Gibco, Thermo Fisher Scientific, Waltham, MA, USA) afterwards. The suspension was then centrifuged for 5 minutes at 2000 rounds per minute (rpm), after which the supernatant was discarded and the pellet resuspended in 5 mL of DNAse medium and 1mL of human serum. After a second centrifugation and discarding of the supernatant, the pellet was resuspended in 2 mL of HCM (90% RPMI 1640 + 10% humane serum (Gibco)). Cells were then incubated overnight at 37°C and 5% CO_2_ to allow the cells to recover. Following an overnight recovery, cells were centrifuged and the remaining pellet was suspended in 3 mL of HCM and the number and viability of cells were assessed. PBMCs were brought to a concentration of 2x10^6^ cells/mL.

### Assessment of Maximal T and B Cell Cytokine Producing Capacity

Maximal cytokine producing capacity was assessed for T and B cells separately by stimulating 1x10^6^ cells/mL PBMCs with a cocktail of phorbol myristate acetate (PMA, 50 ng/mL, Sigma Aldrich, St. Louis, MO, USA) and ionomycin (1 µM, Sigma Aldrich) for 5 hours, of which the last 4 were in presence of the cytokine secretion inhibitor monensin (Golgistop, BD, Erembodegem, Belgium). To control for spontaneous cytokine production, PBMCs were left unstimulated.

After stimulation, frequencies of cytokine producing cells were visualized using a modification of a flow cytometric based assay (44). Briefly, the cell surface was stained using antibodies identifying the different T and B cell subsets and including 7-AAD, a marker to exclude dead cells (**Supplementary Table 1**). T helper cells were identified as CD3+ and CD4+ whereas cytotoxic T cells were identified as CD3+ and CD8+. Within these CD3+CD4+ and CD3+CD8+ T cells, naïve T cells were CCR7+ and CD45RO-, CM T cells were CCR7+CD45RO+, EM T cells were CCR7-CD45RO+ and EMRA T cells were CCR7-CD45RO-. These cells re-express CD45RA and therefore referred to as EMRA T cells. Likewise, the different B cell subsets were identified by expression of CD19. Within these B cells, naïve B cells were CD27-IgD+, non-switched memory B cells were CD27+IgD+, switched memory B cells were CD27+IgD- and the more terminally differentiated B cells were lacking both CD27 and IgD (DN B cells). Upon fixation using BD FACSlysing (BD) and permeabilization using BD FACSPERM II (BD), both according to manufacturer’s instruction, cytokines were stained intracellular using antibodies directed to IL-10, TNF-α, IFN-γ and IL-2 (BioLegend) (**Supplementary Table 2**). Cytokine producing cells were determined by measuring the samples on a BD FACSCanto II (BD) using FACSDiva software version 8 (BD). Analysis of the data was performed using Kaluza Analysis Software version 2.1 (Beckman Coulter, Indianapolis, USA) in order to determine the percentages of T and B cell subsets and frequencies of cytokine producing cells. A typical example depicting the gating strategy applied for analysis of the data is given in (**Supplementary Figures 1A, B**), for T and B cell subsets, respectively. As the number of events for cytokine producing cells within the different subsets was too low, we decided to focus on total CD3+CD4+, total CD3+CD8+ T cells and total CD19+ B cells with respect to proportions of cytokine producing cells.

### Statistical Analysis

Baseline characteristics are reported using descriptive statistics. Comparisons between the three groups (lean controls, morbidly obese patients preoperatively, and morbidly obese patients three months postoperatively) were performed using Pearson’s chi –square test for categorical data, Mann Whitney-U test for unpaired continuous data and the Wilcoxon matched-pairs signed rank test for paired continuous data. Median and range are used as there was no normal distribution of the different variables. The Dirichlet multinomial mixed model was used for statistical analysis of cell subset composition in percentages and frequencies of cytokine producing capacity (45). Adjustment for potential confounders was performed by including interactions for cell type and covariates age, BMI, CMV (yes/no) and MetS (yes/no). BMI was centered at the medians of the respective groups to allow for the selective adjustment of within-group differences in BMI only. Thus, effects due to between-group differences in BMI, e.g. the effect of bariatric surgery, were captured by the indicator variables for the respective groups. Age was centered at the overall median to allow for easier interpretation of the coefficients. The dispersion parameter was modeled as a function of the expected mean. Significance of differences in cell counts was tested by multivariate Wald tests in a sequential fashion. Statistical analysis was performed using Stata version 16.0 (StataCorp, Texas, USA) or R version 3.6.3 (R Foundation for Statistical Computing, Vienna, Austria). Figures were made using Stata version 16.0 (StataCorp, Texas, USA). A two-sided P-value <0.05 was used to indicate statistical significance.

## Results

### Baseline Characteristics

Forty-eight participants were included in this study, consisting of 23 morbidly obese patients and 25 lean controls. Twenty-one morbidly obese patients underwent LRYGB and two underwent LSG. Baseline characteristics are shown in **Table 1**. Twelve (52.2%) morbidly obese patients were clinically diagnosed with MetS preoperatively. The age of the morbidly obese patients was significantly higher than the lean controls (*P*=0.010). Additionally, as expected due to patient selection both weight and BMI of the morbidly obese patients was significantly higher at both measurement points as compared to lean controls (*P*<0.001 for weight and *P*<0.001 for BMI). There was a significant decrease in both weight and BMI three months after bariatric surgery in morbidly obese patients (P<0.001). There was no significant difference in CMV seropositivity between lean controls and morbidly obese patients (P=0.733).

**Table 1:** Study population characteristics.

	Lean controls (n=25)	Morbidly obese patients (n = 23)		*P*-value
		Preoperatively	Postoperatively	
Age (median and range, in years)	29 [25-37]	40 [31-55]		0.010
Weight (median and range, in kg)	73 [68-82]	129 [114.9-140]	106 [90.5-113.8]	<0.001
BMI (median and range, in kg/m^2^)	23.7 [22.4-24.6]	43.4 [38.5-47.9]	34.0 [30.2-37.7]	<0.001
Presence of MetS (number, %)	0 (0%)	12 (52.2%)		<0.001
CMV seropositivity (number, %)	12 (48%)	12 (52.2%)		0.733

BMI, Body Mass Index; MetS, metabolic syndrome; CMV, cytomegalovirus.

### Morbid Obesity Decreased the Cytokine Producing Capacity of CD8+ T Cells as Well as the Single TNF-α Producing Capacity of B Cells

**Figure 1:**
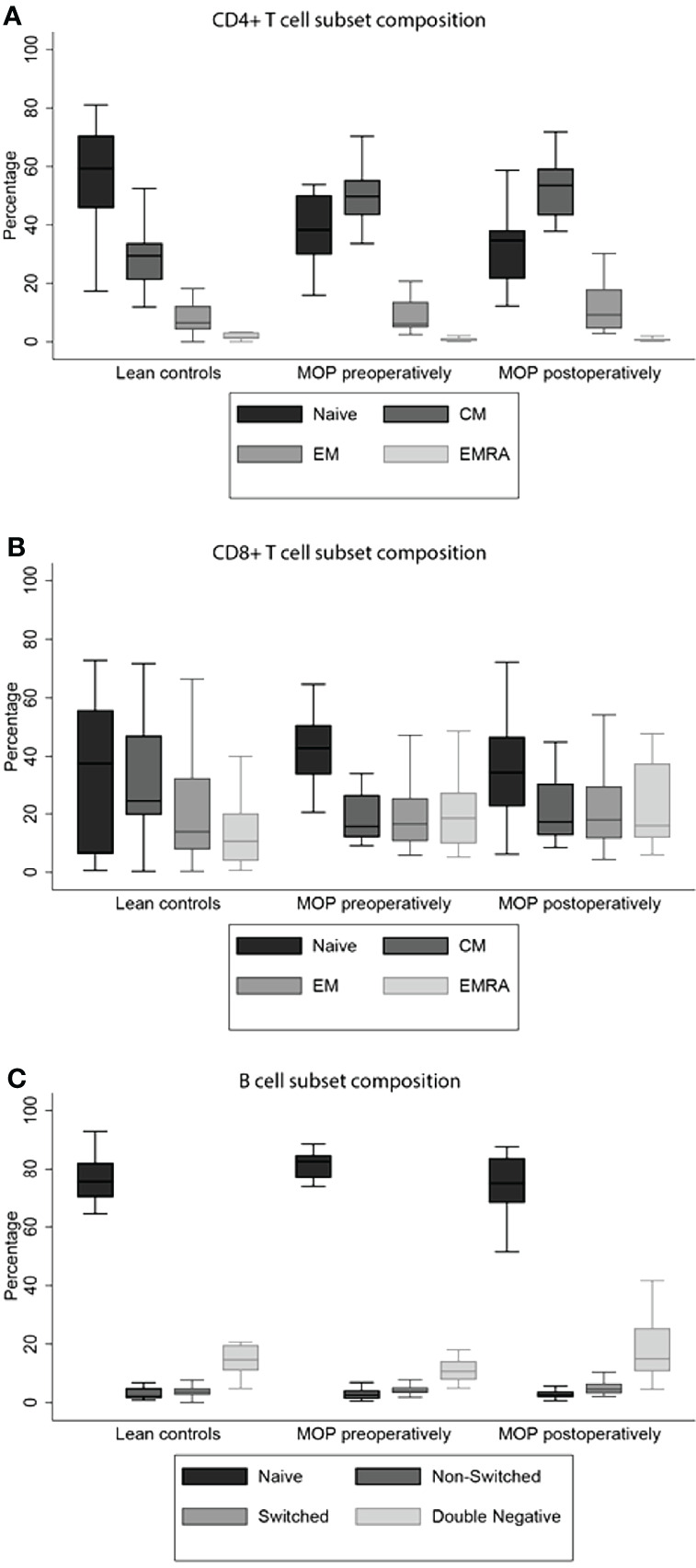
T and B cell subset compositions. Morbid obesity causes a significant decrease in percentage of naive CD4+ T cell **(A)**. The CD8+ T_EMRA_ cells were significantly higher in morbidly obese patients, both preoperatively and postoperatively **(B)**. Both percentages of non-switched and switched B cells were increased in morbidly obese patients **(C)**. Percentages and significances are depicted in **Supplementary Table 3A**. MOP, morbidly obese patients; CM, central memory; EM, effector memory; EMRA, terminally differentiated effector memory.

A statistical difference in CD4+ T cell subset composition was found between lean controls and morbidly obese patients before bariatric surgery (*P*<0.001). Data are depicted in **Supplementary Table 3**. A lower proportion of naïve CD4+ T cells and T_EMRA,_ and higher proportion of T_CM_ was observed in morbidly obese patients before bariatric surgery as compared to lean controls (**Figure 1A**). The total cytokine producing capacity of these cells was not significantly different between the lean controls and morbidly obese patients (**Table 2**).

**Table 2:** Maximal cytokine producing capacity by T cells.

T cell subtype	Specific cytokine producing subset	Lean controls (n = 25)	Morbidly obese patients (n = 23)		*P*-value		
			Preoperatively	Postoperatively	LC vs MOP preoperatively	MOP preoperatively vs MOP postoperatively	LC vs MOP postoperatively
CD4+	Single IL-2 Single IFN-γ IL-2 and IFN-γ	12.5 [9.0-20.0] 6.1 [4.3-8.8] 5.2 [2.6-11.6]	12.8 [7.6-16.6] 5.1 [4.3-7.8] 4.8 [3.0-8.1]	17.1 [9.5-22.3] 7.3 [5.4-10.7] 9.0 [6.3-13.5]	0.851	<0.001	0.103
CD8+	Singe IL-2 Single IFN-γ IL-2 and IFN-γ	4.1 [2.6-8.1] 32.4 [23.4-40.5] 6.2 [2.9-8.8]	2.8 [2.0-5.8] 29.8 [20.4-35.8] 3.6 [1.4-5.2]	3.1 [1.7-5.0] 37.3 [24.9-49.2] 4.0 [1.8-8.5]	<0.001	0.094	0.003

MOP, morbidly obese patients; LC, lean controls; IL-2, interleukin 2; IFN-γ, interferon gamma.

All numbers are presented as percentages of cytokine producing cells in median [interquartile range]. P-values are after correction for covariates using a Dirichlet multinomial mixed model.

The CD8+ T cell subset composition was also significantly different between the lean controls and morbidly obese patients preoperatively (*P*<0.001). This was reflected as a higher proportion of naïve CD8+ T cells and T_EM_ and T_EMRA_, and a lower proportion of T_CM_ in morbidly obese patients (**Figure 1B**). In contrast to CD4+ T cells, the total cytokine producing capacity of CD8+ T cells was significantly different in the morbidly obese patients as compared to lean controls (*P*<0.001) (**Table 2**). Lower proportions of single IL-2, single IFN-γ and IL-2 and IFN-γ producing CD8+ T cells were observed.

The subset composition of B cells was also significantly different (*P*=0.005). The morbidly obese patients had a significantly higher percentage of naïve B cells, while the percentage of DN B cells was lower (**Figure 1C**). There was a difference in the proportions of cytokine producing cells studied (*P*<0.001). The single IL-2 and single IL-10 producing capacity was higher in morbidly obese patients, while the single TNF-α production was lower (**Table 3**). The single IFN-γ producing capacity of B cells was relatively the same between the two different groups.

**Table 3:** Maximal cytokine producing capacity by B cells.

Specific cytokine producing B cell subset	Lean controls (n = 25)	Morbidly obese patients (n = 23)		*P*-value		
		Preoperatively	Postoperatively	LC vs MOP preoperatively	MOP preoperatively vs MOP postoperatively	LC vs MOP postoperatively
Single TNF-α Single IL-2 TNF-α and IL-2	21.9 [15.5-32.3] 1.2 [0.8-1.7] 1.27 [1.0-2.5]	14.8 [11.4-21.0] 3.4 [2.1-5.2] 1.6 [1.0-2.4]	20.2 [13.5-26.4] 4.4 [2.8-6.6] 2.2 [1.1-3.0]	<0.001	0.108	<0.001
Single IL-10 Single IFN-γ IL-10 and IFN-γ	0.5 [0.3-1.0] 2.6 [1.9-6.6] 0.8 [0.5-1.3]	2.8 [1.9-3.6] 2.3 [1.3-2.9] 1.0 [0.7-2.4]	2.9 [2.4-4.3] 2.0 [1.2-2.9] 1.2 [0.7-3.3]	<0.001	0.183	<0.001

MOP, morbidly obese patients; LC, lean controls; TNF-α, tumor necrosis factor-alpha; IL-2, interleukin 2; IFN-γ, interferon-gamma.

All numbers are presented as percentages of cytokine producing cells in median [interquartile range]. P-values are after correction for covariates using a Dirichlet multinomial mixed model.

### Effect of Metabolic Syndrome, Age, CMV Seropositivity Status and BMI on Cytokine Producing Capacity of T and B Cells

**Figure 2:**
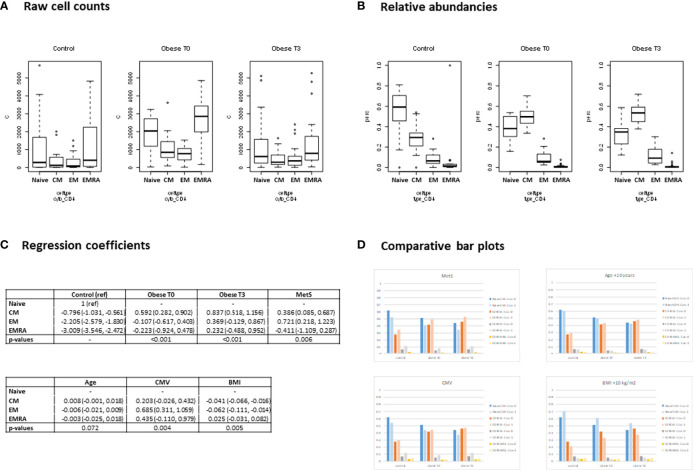
Regression output (Dirichlet multinomial mixed model). CD4 + T cells. **(A)** Raw cell counts - Boxplots showing the observed distribution of the absolute CD4+ T cell counts for healthy controls, and for morbidly obese patients before (T0), and 3 months after bariatric surgery (T3). The cell counts shown are divided by subtype: Naïve; central memory (CM); effector memory (EM); terminally differentiated effector memory (EMRA). **(B)** Relative abundancies - Boxplots showing the observed distribution of relative abundancies of the cell subtypes. For each measurement occasion, the 4 subtypes add up to 100%. **(C)** Coefficients - Using the raw cell counts, a Dirichlet Multinomial Mixed model was fitted modelling the relative abundancies of the cell types. The model was corrected for metabolic syndrome (MetS), Age, CMV status and within group differences in BMI. The regression output is shown here. Coefficients may be interpreted as the log of the relative abundancy compared to the reference cell type (Naive CD4+ T cell). The P-values indicate whether a significant difference in relative abundancy of cell subtypes exists for the morbidly obese patients before and after surgery (Obese T0 and T3) compared to the healthy controls and whether the correcting variables (MetS, Age, CMV and BMI) significantly affect the relative abundancies. **(D)** Comparative bar plots - Based on the regression coefficients, the expected percentages of the 4 cell types were calculated. For each of the 4 correcting variables, the expected percentages are shown when the covariate is present/high (light colored bars) and absent/average (dark colored bars). When comparing the bar plots to the boxplots of the relative abundancies of the raw data, (**Figure 2B**), it can be observed that pattern of the expected percentages based on the model resembles that of the (unadjusted) observed percentages (which is to be expected if the model adequately describes the data).

The effect of covariates was analyzed between the three study groups. For illustrative purposes regression output and inference for CD4 + T cells has been depicted in **Figures 2A–D**. For the regression output of all analyzed cell types, please see **Supplementary Excel Document**. MetS had a significant effect on the relative abundance of cytokine producing T cells for the various cytokines studied (P<0.001). A higher proportion of T cells producing IFN-γ and IL-2 was observed. Presence of MetS also had a significant effect on the cytokine producing capacity of B cells (P=0.004). A higher proportion of single TNF-α producing B cells and a lower proportion of those producing IL-2 was observed.

Age had a significant effect on the relative abundance of cytokine producing CD3+ (P=0.031), CD4+ (P<0.001) and CD8+ (P=0.016) T cells. A higher age tended to lower proportions of single IL-2 producing CD3+, CD4+ and CD8+ T cells. The effect of age on proportions of T cells able to produce IFN-γ was not consistent.

Presence of CMV seropositivity significantly influenced cytokine producing capacity of T cell (P<0.001) and specifically CD8+ T cells (P=0.005), where IL-2 producing capacity tended to be lower and IFN-γ producing capacity tended to be higher.

BMI had a significant effect on the relative abundance of cytokine producing CD4+ T cells (P<0.001), TNF-α and IL-2 producing B cells (P=0.015) and IL-10 and IFN-γ producing B cells (P=0.002). A higher BMI led to a lower proportion of CD4+ T cells to produce IL-2 and IFN-γ as well as a lower proportion B cells producing TNF-α. On the other hand a higher proportion of B cells able to produce IL-2, IL-10 and IFN-γ was observed.

### Bariatric Surgery Does Not Improve the Cytokine Producing Capacity of T and B Cells

After bariatric surgery, the CD4+ T cell subset composition significantly changed (*P*=0.013). This difference was caused by a decrease of the percentage naïve CD4+ T cells, while the percentages of T_em_, T_CM_ and T_EMRA_ cells increased (**Supplementary Table 3**). However, the subset composition was not reversed towards that of lean controls. Notably, bariatric surgery increased the single as well as total IL-2 and IFN-γ producing capacity of the CD4+ T cells in morbidly obese patients, which was comparable to the cytokine producing capacity of the lean controls (*P*=0.103) (**Table 2**).

The CD8+ T cell composition was not influenced by bariatric surgery (*P*=0.186). Particularly, the CD8+ T cell subset composition after bariatric surgery remained significantly different as compared to the lean controls (*P*=0.019) (**Supplementary Table 3**). In contrast to the CD4+ T cell cytokine producing capacity, the CD8+ T cell cytokine producing capacity was not altered by bariatric surgery (*P*=0.094). However, there was a slightly higher proportion of CD8+ T cells producing IFN-γ and a lower proportion producing IL-2 after bariatric surgery compared to that measured for lean controls (**Table 2**).

After bariatric surgery, the B cell subset composition adjusted towards the profile of lean controls (**Supplementary Table 4**). Even though the composition of the B cell compartment was reversed by bariatric surgery, the cytokine producing capacity was not. When comparing the cytokine producing capacity of morbidly obese patients after bariatric surgery to that of lean controls, it remained significantly different, i.e. comparable to the difference between morbidly obese patients before bariatric surgery and lean controls (**Table 3**).

## Discussion

In this study, we compared a cohort of morbidly obese patients to that of lean controls, and evaluated the effects of bariatric surgery with respect to composition and cytokine producing capacity of T and B cells. The main findings include a shift towards a more differentiated CD4+ and CD8+ T cell compartment in morbidly obese patients as compared to lean controls; three months after bariatric surgery, this had not changed towards the profile of lean controls. The IL-2 and IFN-γ producing capacity of CD8+ T cells was significantly decreased by morbid obesity, which was not influenced by bariatric surgery. The B cell subset composition of morbidly obese patients adjusted towards the profile of lean controls three months after bariatric surgery. Nonetheless, the cytokine producing capacity of these cells was not reversed by bariatric surgery.

The decrease in naive CD4+ T cells in morbidly obese patients is similar to previously described findings in mice (16, 20). The chronic inflammation in morbid obesity might lead to accelerated aging of the immune system (46, 47). In our study, we found an increase in T_CM_ CD4+ T cells, while the T_EM_ CD4+ T cells seemed unaffected. Although the findings were significantly different, the clinical implication is debatable as the difference was just one percent. In contrast to our study, a study performed in morbidly obese individuals described an increase in both naive and memory T cells (15). In this study, this increase in naive T cells was explained as a reaction to the antigenic load. An alternative explanation for the increase of naive T cells is that the thymic production of T cells is not affected by morbid obesity.

We found an increase in naive B cells, which has been described before and can be explained by the chronic inflammation in morbidly obese patients (10, 48). This chronic inflammation causes mobilization of developing B cells from the bone marrow into peripheral immune organs and peripheral blood. There was a decrease in DN B cells, which is in contrast to what we expected. We expected that the chronic low-grade inflammation in morbidly obese patients would lead to an increase in the more differentiated DN B cells (10). A possible explanation for this finding is that there was such a large increase of naïve B cells, that the DN B cells in the B cell composition in percentages decreased. Nevertheless, much remains unclear about the origins of the differences in B cell subset composition, and further research into this topic is therefore recommended.

Jongbloed et al. described enhanced CD8+ T cell differentiation in morbidly obese patients, which was mainly related to the presence of MetS (43). The CD8+ T cell differentiation state was comparable to what we found, however, CD8+ T cell differentiation was not affected by MetS. These different findings can be explained by the larger population of morbidly obese patients and thus bigger subpopulations. Even so, the presence of MetS seemed to increase the IFN-γ and IL-2 producing capacity of CD4+ T cells and the TNF-α producing capacity of B cells, and to decrease the IL-2 producing capacity of B cells. Bariatric surgery had an effect on the producing capacity of CD4+ T cells, but not on that of CD8+ T cells and B cells. In literature, it is suggested that changes in cellular immunity after weight loss is linked to metabolic improvement (49). As our follow-up period was three months, not all patients with MetS prior to surgery might have recovered from this during the short follow-up period. This might explain why the cytokine producing capacity of CD8+ T cells and B cells was not restored in our study.

We found an increase in proportion of IL-2 and IFN-γ producing CD4+ T cells. This stands in contrast to an earlier published article by Zhan et al., in which follicular helper T cells were identified (37). Three months after LRYGB, the follicular helper T cells had an altered function, resulting in a decrease in IFN-γ, IL-2, IL-4 and IL-17 secretion and an increase of IL-10 secretion. This study included a total of eight patients and all patients were diagnosed with T2D, whereas only six patients (26%) in our study were diagnosed with T2D preoperatively. Lips et al. showed an increase in TNF-α secretion by T cells three months after LRYGB (50). However, they have not investigated the B cell cytokine producing capacity.

One of the limitations of this study is the follow-up period of three months. Although weight loss follows rapidly after bariatric surgery, the total expected excess weight loss after bariatric surgery is typically achieved after twelve to eighteen months (51). Moreover, the immune system might need a longer period to recover from the alterations caused by morbid obesity. In a previous study, a decrease in CD8+ T_EM_ cells was found three months after bariatric surgery, while a decrease of CD4+ T_EM_ and T_EMRA_ was found six months after bariatric surgery (43). These findings indicate that alterations of the immune system caused by morbid obesity could be restored after bariatric surgery, but that some changes are reached after a longer period of time. We would therefore suggest additional research into the effect of bariatric surgery on the immune system for a follow-up period of at least eighteen months. Another limitation of this study is the study population. Although our study is larger than most other studies (36, 37), a larger cohort could lead to a better distinction between the observed differences between the study groups. By this, the influence of MetS on the immune system can be investigated more thoroughly. As an increased age leads to a more differentiated subset composition, age-matched controls are recommendable. As we received the blood collections from the Sanquin blood bank, we could not obtain samples in the exact same age. However, we performed a mixed model analysis with correction for age, and age only influenced the cytokine producing capacity of T cells. Unfortunately, the gender of blood bank donors was unknown. Therefore, the influence of gender on the outcomes could not be assessed. However, previous research from Jongbloed et al. shows that gender did not affect the results (37). Thus it can be expected that gender did not affect the results in this study either. Besides this, it would be interesting to compare the postoperative data with lean controls who have undergone surgery as well. Furthermore, the analysis of the immune system in this study was performed on lymphocytes from the peripheral blood. Some studies have reported effects of morbid obesity in T and B cells in adipose tissue, such as an increase of proinflammatory cytokine production by adipose-resident T cells and a decrease of IL-10 production by B cells (20, 52). It would therefore be interesting to compare the T and B cell subset composition and function in both peripheral blood and the adipose tissue. In the present study, we have only studied the cytokine producing capacity of six cytokines. It would be interesting to expand the studied cytokines. Furthermore, it would be interesting to investigate the vaccination response of morbidly obese patients before and after bariatric surgery, as this is indicative for the quality of the immune system. We analyzed the effect of CMV seropositivity status on results because this might affect the cytokine producing capacity of immune cells. Even though covariate analysis showed a potential negative effect of CMV seropositivity, sample size was too small to analyze positive and negative subjects separately. However, this finding would be an interesting issue for further research in a larger study population. Since we only assessed morbidly obese patients in our study, we cannot extrapolate results to obesity in common. It would be interesting to investigate whether the alterations we found, also appear in non-morbidly obese individuals. However, these patients do not meet criteria for bariatric surgery in our hospital and could therefore not be included in our study.

Our data suggest accelerated differentiation of CD4+ and CD8+ T cells in morbidly obese patients as compared to lean controls. Even though this did not influence the cytokine producing capacity of CD4+ T cells, the IL-2 and IFN-γ production of CD8+ T cells was decreased in morbidly obese patients. Bariatric surgery changed CD4+ T cell and B cell subset composition towards the profile of lean controls, and the IL-2 and IFN-γ producing capacity of CD4+ T cells was increased three months after bariatric surgery. However, the cytokine producing capacity of CD8+ T cells and B cells was not restored three months after bariatric surgery. A longer follow-up period after bariatric surgery is recommended as the immune system might need more than three months to recover from immune changes caused by morbid obesity, as well as patients might need more time to recover from MetS.

Altogether, these data suggest that the composition of the immune system has the capacity to recover from the detrimental effects of morbid obesity after bariatric surgery, but the cytokine producing capacity of T and B cells is not recovered after 3 months.

## Data Availability Statement

The original contributions presented in the study are included in the article/**Supplementary Material**. Further inquiries can be directed to the corresponding author.

## Ethics Statement

The studies involving human participants were reviewed and approved by Medical Research Ethics Committees United (MEC-U). The patients/participants provided their written informed consent to participate in this study.

## Author Contributions

LW, EH, RK, FJ, RB and NL contributed to the design and implementation of the research. LW, GA and MK carried out the experiments. LW, AT and TK performed the analysis of the results. LW, AT and FN took the lead in writing the manuscript in consultation with RB. and NL. All authors discussed the results and commented on the manuscript.

## Funding

The materials for immune cell phenotyping were funded by Medtronic, Netherlands.

## Conflict of Interest

The authors declare that the research was conducted in the absence of any commercial or financial relationships that could be construed as a potential conflict of interest.

## Publisher’s Note

All claims expressed in this article are solely those of the authors and do not necessarily represent those of their affiliated organizations, or those of the publisher, the editors and the reviewers. Any product that may be evaluated in this article, or claim that may be made by its manufacturer, is not guaranteed or endorsed by the publisher.
