# Supplementary material for: T and B Cell Composition and Cytokine Producing Capacity Before and After Bariatric Surgery
Source: Front Immunol. 2022 Jul 4;13:888278. doi: 10.3389/fimmu.2022.888278 (PMC9289114; doi:10.3389/fimmu.2022.888278)
Supplement: Supplementary file 1 [file DataSheet_1.docx]

**Supplementary Table 1. Antibodies for surface staining**

| ***T cells*** | | | | |
| --- | --- | --- | --- | --- |
| **Antibody** | **Clone** | **Dilution** | **Titer/100 µl cell suspension** | **Firm** |
| CD3 BV510 | OKT3 | 1:10 | 10 µl | Biolegend |
| CD4 PacBlue | RPA-T4 | 1:40 | 10 µl | Biolegend |
| CD8 APC-Cy7 | SK1 | 1:40 | 10 µl | Biolegend |
| CD45RO APC | UCHL1 | 1:10 | 10 µl | Biolegend |
| CCR7 PE-Cy7 | G043H7 | Undiluted | 5 µl | Biolegend |
| 7AAD |  | Undiluted | 5 µl | Biolegend |
| ***B cells*** | | | | |
| **Antibody** | **Clone** | **Dilution** | **Titer/100 µl cell suspension** | **Firm** |
| CD19 BV510 | HIB19 | Undiluted | 5 µl | Biolegend |
| CD27 PE-Cy7 | O323 | 1:40 | 10 µl | Biolegend |
| IgD APC-Cy7 | IA6-2 | 1:10 | 10 µl | Biolegend |
| CD38 BV421 | HIT2 | 1:10 | 10 µl | BD |
| CD24 APC | ML5 | Undiluted | 2 µl | Biolegend |
| 7AAD |  | Undiluted | 5 µl | Biolegend |

**Supplementary Table 2. Antibodies for intracellular staining**

| ***T cells*** | | | | |
| --- | --- | --- | --- | --- |
| **Antibody** | **Clone** | **Dilution** | **Titer/100 µl cell suspension** | **Firm** |
| IL2 FITC | MQ1-17H12 | 1:10 | 10 µl | Biolegend |
| IFN-γ PE | B27 | 1:20 | 10 µl | Biolegend |
| ***B cells mix 1*** | | | | |
| **Antibody** | **Clone** | **Dilution** | **Titer/100 µl cell suspension** | **Firm** |
| IL2 FITC | MQ1-17H12 | 1:10 | 10 µl | Biolegend |
| TNF-α PE | MAb11 | Undiluted | 2 µl | Biolegend |
| ***B cells mix 2*** | | | | |
| **Antibody** | **Clone** | **Dilution** | **Titer/100 µl cell suspension** | **Firm** |
| IL10 PE | JES3-9D7 | Undiluted | 5 µl | Biolegend |
| IFN-γ FITC | 4S.B3 | Undiluted | 5 µl | Biolegend |

**Supplementary Table 3. T cell subset compositions in percentages**

| **T cell subtype** | **Lean controls (n=25)** | **Morbidly obese patients (n=23)** | | ***P*-value** | | |
| --- | --- | --- | --- | --- | --- | --- |
|  |  | **Preoperatively** | **Postoperatively** | **LC vs MOP preoperatively** | **MOP preoperatively vs MOP postoperatively** | **LC vs MOP postoperatively** |
| CD4+ naive  CD4+ CM  CD4+ EM  CD4+ EMRA | 59.2 [45.7-70.7]  29.4 [21.1-33.8]  6.5 [4.1-12.3]  1.6 [0.9-3.3] | 38.2 [29.8-50.2]  49.7 [43.3-55.4]  6.1 [4.8-13.7]  0.4 [0.2-1.3] | 34.7 [21.5-38.3]  53.5 [43.2-59.3]  9.2 [4.5-18.0]  0.5 [0.2-1.0] | <0.001 | 0.013 | <0.001 |
| CD8+ naive  CD8+ CM  CD8+ EM  CD8+ EMRA | 37.6 [6.3-55.9]  24.2 [19.6-47.2]  13.9 [7.8-32.6]  10.6 [3.9-20.3] | 42.8 [33.8-50.8]  15.7 [12.0-26.5]  16.6 [10.6-25.4]  18.5 [9.8-27.3] | 34.5 [22.5-46.8]  17.2 [12.7-30.4]  17.9 [11.6-29.4]  15.9 [11.8-37.7] | <0.001 | 0.186 | 0.019 |

MOP = morbidly obese patients; LC = lean controls; CM = central memory; EM = effector memory; EMRA = terminally differentiated effector memory.
All percentages are presented as median [interquartile range]. *P*-values are after correction for covariates using a Dirichlet multinomial mixed model.

**Supplementary Table 4. B cell subset compositions in percentages**

| **B cell subtype** | **Lean controls (n=25)** | **Morbidly obese patients (n=23)** | | ***P*-value** | | |
| --- | --- | --- | --- | --- | --- | --- |
|  |  | **Preoperatively** | **Postoperatively** | **LC vs MOP preoperatively** | **MOP preoperatively vs MOP postoperatively** | **LC vs MOP postoperatively** |
| Naive  Non-switched  Switched  Double negative | 75.4 [70.0-82.2]  2.2 [1.5-5.0]  3.7 [2.7-4.9]  14.6 [10.9-19.7] | 82.6 [76.6-84.8]  2.6 [1.3-4.3]  4.0 [3.3-5.3]  10.7 [7.7-14.2] | 74.7 [68.1-83.8]  2.5 [1.9-3.7]  4.5 [3.1-6.6]  14.9 [10.6-25.5] | 0.005 | <0.001 | 0.095 |

MOP = morbidly obese patients; LC = lean controls

All percentages are presented as median [interquartile range]. *P*-values are after correction for covariates using a Dirichlet multinomial mixed model.

**Supplementary Figure 1. Typical examples of the flow cytometric gating strategies**

1. **Flow cytometric gating strategy for CD4+ and CD8+ T cell subsets and frequencies of cytokine producing cells**

First, lymphocytes were gated and then viable CD3+ T cells were selected. From these viable T cells, CD4+ or CD8+ T cells were selected. The CD4+ and CD8+ T cells were then subdivided into the main T cell subsets based on the expression of CCR7 and CD45R0, with CD45R0^-^CCR7^+^ being naïve T cells, CD45R0^+^CCR7^+^ central memory T cells (CM), CD45R0^+^CCR7^-^ effector memory (EM) and CD45R0^-^CCR7^-^ terminally differentiated effector memory (EMRA) T cells. Additionally, CD4+ and CD8+ T cells were subdivided based on the expression of IL-2 and/or IFN-γ.

1. **Flow cytometric gating strategy for B cell subsets and frequencies of cytokine producing cells**

First, lymphocytes were gated and then viable CD19+ B cells were selected. These B cells were then subdivided into B cell subsets based on the expression of CD27 and IgD, with CD27-IgD+ being naive, CD27+IgD+ non-switched, CD27+IgD- switched and CD27-IgD- double negative (DN) B cells. Furthermore, B cells were subdivided based on the expression of TNF-α and/or IL-2 and of IFN-γ and/or IL-10.
