# Supplementary figures and images for: T and B Cell Composition and Cytokine Producing Capacity Before and After Bariatric Surgery
Source: Front Immunol. 2022 Jul 4;13:888278. doi: 10.3389/fimmu.2022.888278 (PMC9289114; doi:10.3389/fimmu.2022.888278)

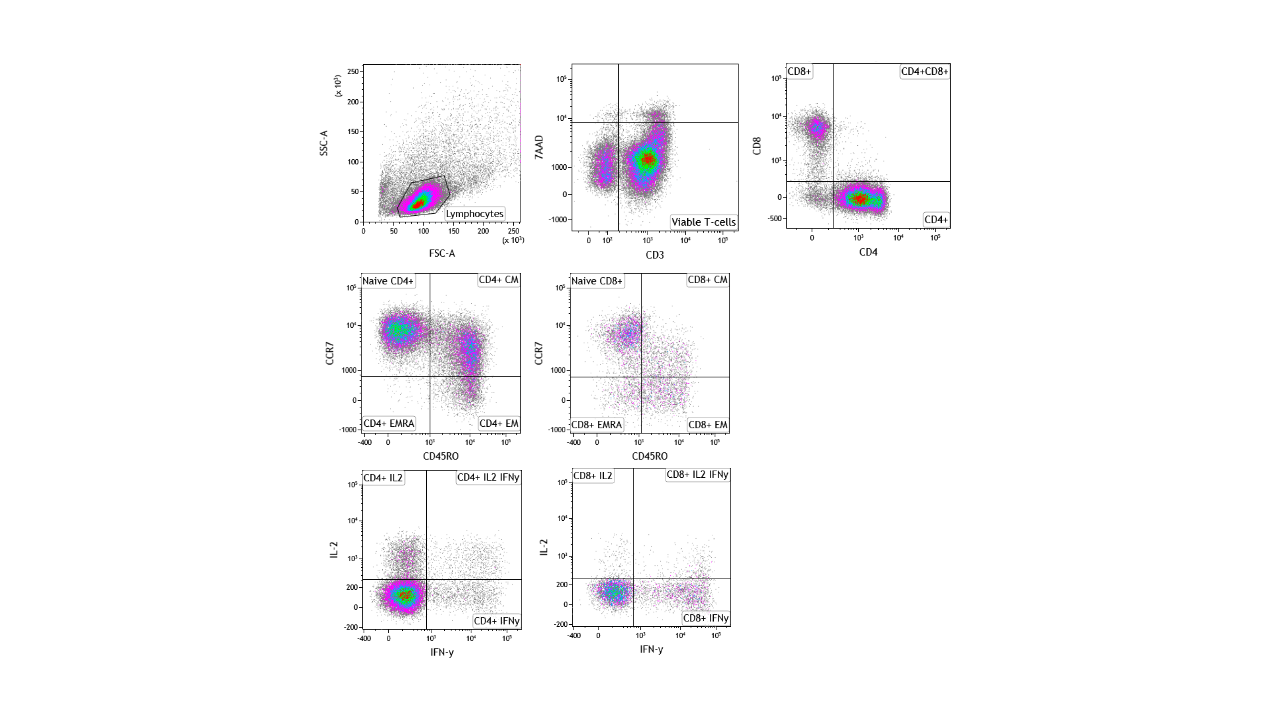

Supplement: Supplementary file 3 [file Image_1.tif]

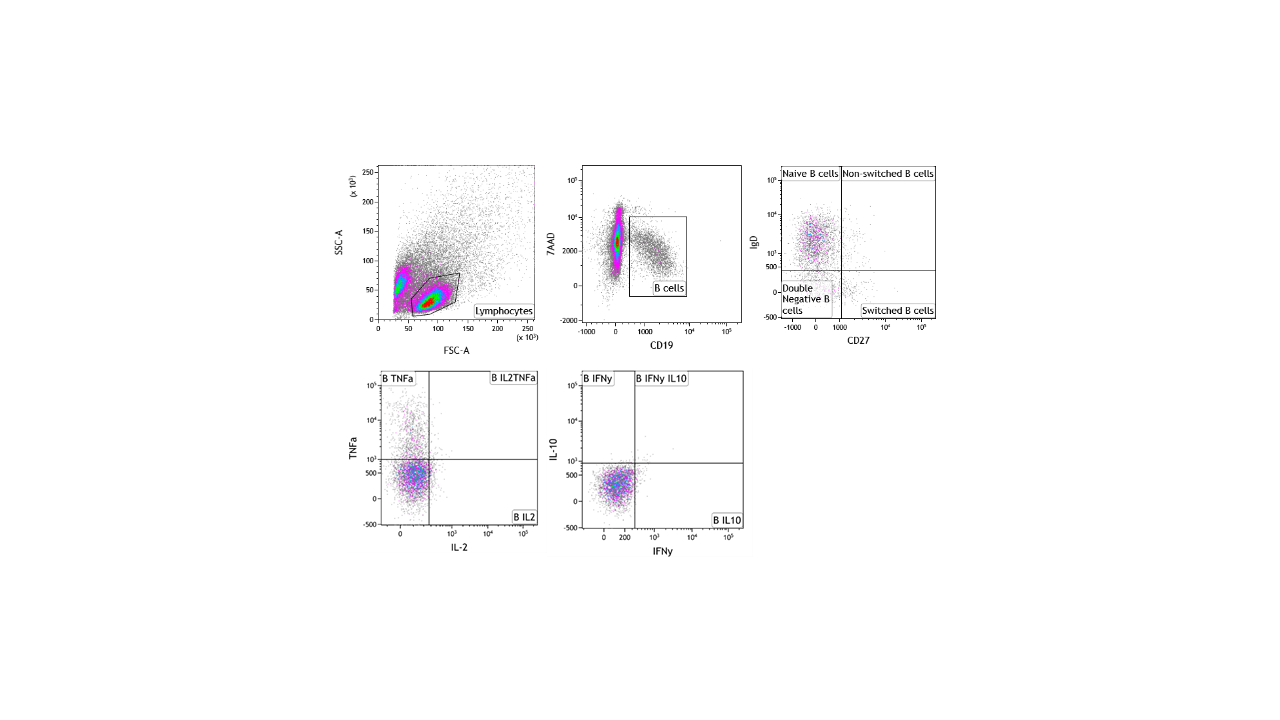

Supplement: Supplementary file 4 [file Image_2.tif]
